# Isolated thyroid metastasis revealed an unknown lung adenocarcinoma: a case report

**DOI:** 10.1186/s13256-015-0663-z

**Published:** 2015-09-27

**Authors:** J. Khalil, F. Elomrani, M. Benoulaid, H. Elkacemi, T. Kebdani, H. Errihani, N. Benjaafar

**Affiliations:** 1 Radiation oncology department, National Institute of oncology, Mohamed V University, Rabat, Morocco; 2 Medical oncology department, Mohamed V University, Rabat, Morocco

**Keywords:** Thyroid metastasis, primitive lung carcinoma, rare association

## Abstract

**Introduction:**

Cancer metastasis to the thyroid is extremely rare. The most common sites that have been reported to metastasize to the thyroid gland are breast and kidney. As to primary lung cancer metastasizing to the thyroid gland, only a few cases have been described in the literature.

**Case presentation:**

We report a case of a 37-year-old white Arabian woman who had never smoked tobacco products for whom a malignant thyroid mass revealed a primary lung tumor. She had a surgical excision for both the thyroid and the pulmonary tumors, and received adjuvant chemotherapy. At 1 year, she is still in remission.

**Conclusions:**

Our case is rare as it describes a case where the thyroid lesion was the revealing sign of an unknown lung carcinoma. Management of thyroid metastases should depend on the individual situation and surgical excision should be proposed whenever a patient’s condition is favorable.

## Introduction

The thyroid gland is a rare target for metastatic tumors, despite its rich vascular supply [1]. According to autopsy studies, the incidence of metastatic tumors to the thyroid ranges from 3.9% to 24.2% [2, 3]. The reported clinical incidence does not exceed 4% of all thyroid malignancies [4]. The breast and kidney are the most reported primary sites to metastasize to the thyroid gland. Isolated lung metastatic disease to the thyroid gland has been rarely reported in the literature [4–15]. To the best of our knowledge, only a few cases of metastatic lung adenocarcinoma to the thyroid gland have been reported, describing all together four cases of thyroid lesions revealing a lung carcinoma. In our clinical case, the thyroid was the site of an unusual metastasis revealing an underlying lung adenocarcinoma not initially suspected.

## Case presentation

A 37-year-old white Arabian woman who had never smoked tobacco products presented a nodular goiter during a routine follow up with her endocrinologist. An ultrasound examination of her neck showed a 2.4×3.2cm solid hypoechoic nodule of her right thyroid lobe. Her left thyroid lobe and the isthmus appeared to be normal. Serum levels of free triiodothyronine, free thyroxine, and thyrotropin were within normal ranges. She was screened for multiple endocrine neoplasias with negative results.

She underwent ultrasonogram-guided fine-needle aspiration cytology (FNAC) that showed a cellular aspirate comprising predominantly atypical epithelial cells having hyperchromatic eccentric nucleus and a moderate to abundant amount of vacuolated cytoplasm with occasional scattered thyroid follicular epithelial cell clusters with absence of colloid.

Her chest X-ray and abdominal ultrasound were normal.

**Fig. 1:**
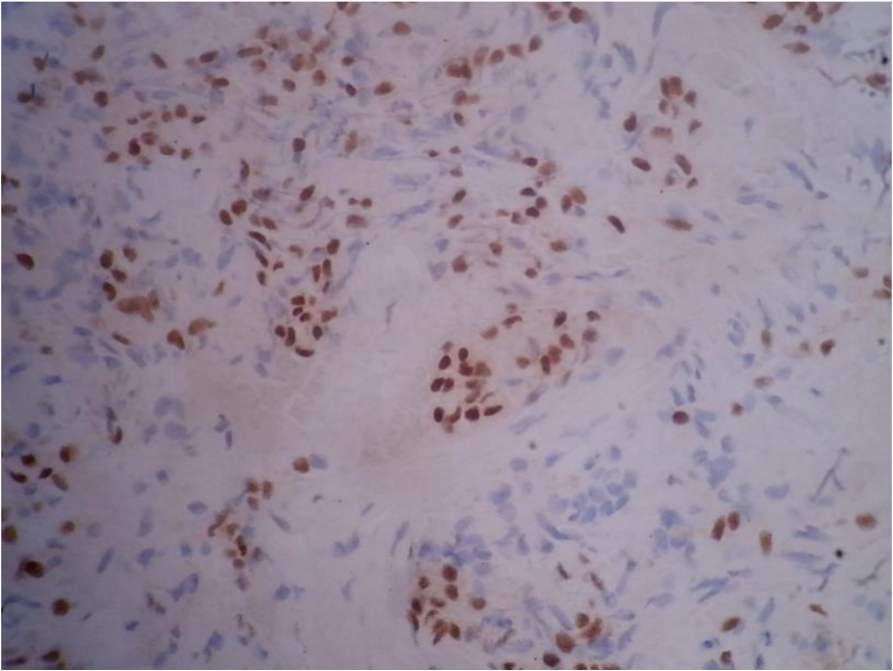
High power view demonstrating positive immunostaining for thyroid transcription factor-1 (G ×40)

**Fig. 2:**
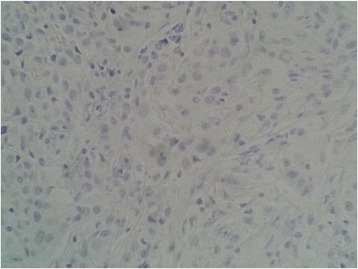
High power view demonstrating negative immunostaining for thyroglobulin (G ×40)

She underwent a total thyroidectomy with neck exploration. The microscopic aspect of the nodule was consistent with a well-differentiated carcinoma. Immunohistochemistry demonstrated that these tumor cells were positive for both cytokeratin 7 (CK7) and thyroid transcription factor-1 (TTF-1) and were negative for both CK20 and thyroglobulin, which concords with metastatic disease from lung origin (Figs. 1 and 2).

A computed tomography (CT) scan of her chest along with an upper abdomen CT was then performed, and revealed a 4cm mass within the right upper lobe of her lung. Radiologic findings were negative for both lymph node and adrenal metastases.

Given these results, our case was presented during a weekly board meeting, and the decision of a surgical excision of the pulmonary mass was made. Three weeks later, our patient underwent a segmentectomy with lymph node dissection. According to the pathologic findings, the tumor was staged as IB (pT2a,N0,M0). She was referred to our medical oncology department for adjuvant therapy, where she received six cycles of an adjuvant platinum and vinorelbine-based regimen.

After 1 year of follow up, she continues to be in remission.

## Discussion

Lung cancer is the leading cause of cancer death worldwide, with an estimated 1.4 million deaths each year [16]. Lung cancer also has a high prevalence of metastases; it commonly metastasizes to the brain, bones, adrenal glands, contralateral lung, and liver. Despite the rich vascular supply of the thyroid gland, coming right after the adrenals [17], it has been rarely reported as a site of metastases especially to lung carcinoma.

The incidence of thyroid metastases in patients with a known malignancy varied in large autopsy series from 1.9 to 24% [18–21]. In these series, cancers of the breast, lung and malignant melanoma were the most frequent malignancies to metastasize to the thyroid, with the metastases being clinically occult. The site of origin of clinically significant thyroid metastases appears to be different. In fact, many clinical series have shown that renal cell carcinoma is the most common primary tumor causing symptomatic thyroid metastases, followed by breast and lung [22–27].

It is often difficult to distinguish between metastasis and a second primary cancer. Typically, the interval between the diagnosis of the primary tumor and the detection of thyroid metastasis is from 1 month to 26 years [4, 17]. In our case, a thyroid lesion was the revealing sign of lung adenocarcinoma; only a handful of similar cases have been described in the literature [7, 8].

In our case, the diagnosis of the thyroid metastasis was based on FNAC, which was further substantiated by the biopsy and immunohistochemical examination (IHC). Fine-needle aspiration (FNA) with ultrasound guidance is a rapid, minimally invasive, and inexpensive technique with a high predictive negative value [28]. In the metastatic setting, there is abundant cellularity and the cells may be typical of the original site, especially when specific immunohistochemical stains are performed. Negative staining with anti-thyroglobulin and anti-calcitonin antibodies would favor a metastatic tumor. However, FNA may not be contributory to the diagnosis as it may contain insufficient tumor cells to make the cell block used for immunostains. Diagnostic re-evaluation of the primary tumor and search for other metastatic sites is necessary.

Metastases to the thyroid gland were associated with a poor prognosis in multiple series [1]. However, radical treatment of an isolated metastasis to the thyroid gland was shown to be curative [4, 21]. In fact, an improvement in the survival rates (34 months of median survival versus 25 months in the not operated group) was reported when radical treatment was employed [5]. Another series, reported a disease-free survival of 84 months in the patients where lobectomy was performed [10]. Thus, an aggressive surgical approach has been recommended by many authors [8, 29–32], even though surgical treatment is still controversial. Papi *et al*. [27], in their report suggested that the good indication of thyroidectomy for multinodular toxic goiter (MTG) is limited to patients whose metastases are limited only to the thyroid from the viewpoint of preventing further dissemination of the primary tumor. External beam radiation therapy (EBRT) has also been described as a good therapeutic approach for palliation of symptoms due to thyroid metastases [8]. In our case, the patient had a radical excision of both the primary tumor and the metastasis, and remains well after a 1-year follow up.

Given the available data, we can conclude that the survival of patients with thyroid metastases is variable and depends on the primary cancer, the treatment approach and the presence of other distant metastases. Primary renal cancer has been reported to be associated with better survival rates when compared to extrarenal locations [19]. Prolonged survival of more than 5 years was observed in the cases where surgical excision was performed [4, 7, 17, 28]. Finally, patients with multiple metastases present the worse survival rates (5% at 5 years) [33].

## Conclusions

Metastases to the thyroid gland are not as uncommon as previously thought; however, our case is unusual as it describes a case where the thyroid lesion was the revealing sign of an unknown lung carcinoma. Metastases to the thyroid gland have been associated with poor outcomes, although surgical treatment enhances the chances of better overall survival and disease-specific survival. Therefore, management of thyroid metastases should depend on the individual situation and surgical excision should be proposed whenever a patients’ condition is favorable.

## Consent

Written informed consent was obtained from the patient for publication of this case report and any accompanying images. A copy of the written consent is available for review by the Editor-in-Chief of this journal.

## Abbreviations

CT: Computed tomography
FNA: Fine needle aspiration
FNAC: Fine needle aspiration cytology
